# Ti_3_C_2_ mediates the NiFe-LDH layered electrocatalyst to enhance the OER performance for water splitting

**DOI:** 10.1016/j.heliyon.2024.e30966

**Published:** 2024-05-14

**Authors:** Yaxun Sun, Ze Wang, Qianyu Zhou, Xin Li, Dongye Zhao, Bo Ding, Shifeng Wang

**Affiliations:** a Key Laboratory of Plateau Oxygen and Living Environment of Tibet Autonomous Region, College of Science, Tibet University, Lhasa, 850000, China; b College of Information Engineering, Xizang Minzu University, Xianyang 712000, China

**Keywords:** Layered double hydroxides, OER, Electrocatalyst, Ti_3_C_2_, 2D materials

## Abstract

Oxygen evolution reaction (OER) is a very complex process with slow reaction kinetics and high overpotential, which is the main limitation for the commercial application of water splitting. Thus, it is of necessary to design high-performance OER catalysts. NiFe based layered double hydroxides (NiFe-LDHs) have recently gained a lot of attention due to their high reaction activity and simple manufacturing process. In this study, a novel electrocatalyst based on NiFe-LDH was constructed by introducing Ti_3_C_2_, which was utilized to modulate the structural and electronic properties of the electrocatalysts. Structural examinations reveal that the Ti_3_C_2_ of 2D structure successfully dope the NiFe-LDHs nanosheets, forming NiFe-LDH/Ti_3_C_2_ heterojunctions. Firstly, the heterojunction substantially reduces the charge transfer resistance, promoting the electron migration between the LDH nanosheets. Secondly, theoretical calculations demonstrate that the energy barrier between the rate-determining step from *OH to *O is lowered, favoring the formation of the reaction intermediates and thus the occurrence of OER. As a result, the composite electrocatalyst exhibits a low overpotential of 334 mV at a current density of 10 mA/cm^2^ and a small Tafel slope of 55 mV/dec, which are superior to those of the NiFe-LDH by 11.2 % and 38.5 %, respectively. This study provides inspiration for promoting the performances of NiFe based electrocatalysts by utilizing 2D materials.

## 1 Introduction

**Scheme 1:**
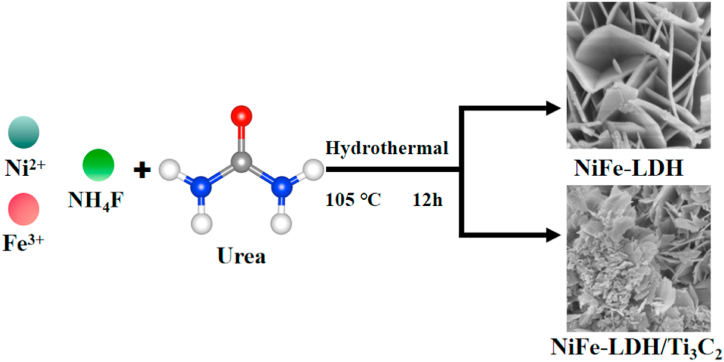
Preparation process of NiFe-LDH and NiFe-LDH/Ti_3_C_2_.

To curb pollution and the energy crisis, water electrolysis is a key factor in clean and renewable energy technologies [[1], [2], [3], [4]]. The oxygen evolution reaction (OER) reaction kinetics is slow, due to its high overpotential. Developing new catalytic materials for oxygen evolution is an important way to improve the efficiency of water electrolysis in new energy [5,6]. At present, the most efficient catalytic materials for OER are iridium, ruthenium oxides (IrO_2_, RuO_2_) and other noble metals. However, its scarce reserves and expensive prices limit large-scale commercial application. Therefore, the design high-performance OER catalysts are critical, but still challenging [[7], [8], [9]] (see Scheme 1)

Layered double metal hydroxides (LDHs) are metal hydroxides composed of two or more metal elements. Due to the advantages of structure tailoring and functionalization with other materials, the composition can be easily adjusted. LDHs are widely used in various applications such as catalysts, adsorbents, and ion exchangers. The catalyst local structure can be adjusted by partially or completely transforming into a new structure with an active phase, which boosts the performance of the active components. Hence, it is necessary to design LDH-based OER catalyst [[10], [11], [12], [13], [14], [15], [16]]. Lou et al. reported an ultrathin Ni–Fe LDH nanosheets with large surface areas display high electrocatalytic activity towards the OER, which exhibited a low overpotential, a small Tafel slope, and remarkable stability [17]. Zhang et al. reported non-noble metal based 3D porous electrocatalyst, which is prepared by self-assembly of the liquid-exfoliated single-layer CoAl-layered double hydroxide nanosheets (CoAl-NSs) onto 3D graphene network. The catalyst exhibits higher catalytic activity and better stability for electrochemical oxygen evolution reaction compared to the commercial IrO_2_ nanoparticle-based 3D porous electrocatalyst [18]. Geng et al. reported FeNi-LDHs arrays on the Ni foamfabricated by a two-step hydrothermal approach with a rougher surface and larger active area than the one-step hydrothermal method, which showed efficient electrocatalysts for overall water splitting [19].

However, the intrinsic electrical conductivity of NiFe LDH is relatively poor, which affects the rate of electron transfer during the catalytic process [[20], [21], [22], [23], [24]]. Ti_3_C_2_, as a typical two-dimensional transition metal carbide, is considered an ideal composite material due to its excellent electrical conductivity, outstanding chemical stability, and large active surface area [25,26]. Firstly, the introduction of Ti_3_C_2_ can serve as a high-speed channel for electron transfer, reducing the internal transfer impedance of electrons within NiFe LDH, thereby enhancing the overall charge transfer rate [27]. Secondly, the layered structure of Ti_3_C_2_ may be compatible with the layered structure of NiFe LDH, which helps to maintain structural stability after compounding, a factor that is crucial for the long-term operation of catalytic reactions [28]. Lastly, the combination of NiFe LDH with Ti_3_C_2_ can produce a synergistic effect, improving the catalytic activity of NiFe LDH through interfacial actions [29].

In this work, incorporating Ti_3_C_2_ into transition metal LDHs is an effective method to design high-performance OER catalyst. Ti_3_C_2_ was used to modulate the electrical and electronic properties of the NiFe-LDH electrocatalyst, electrically lowering the charge transfer resistance and energetically favoring the OER process. The composite catalyst exhibits a substantially lower overpotential and a smaller Tafel slope than that of the pure NiFe-LDH, which shows great improvements compared with the pristine NiFe-LDHs and demonstrates excellent potential for industrial application.

## 2 Materials and methods

### 2.1 Materials

Analytical-grade nickel nitrate hexahydrate (Ni(NO_3_)_2_·6H_2_O) was bought from Damao Chemical Reagent Factory. Iron (III) nitrate nonahydrate (Fe(NO_3_)_3_·9H_2_O, 99.99 %) and ammonium fluoride (NH_4_F, 99.99 %) were obtained from West Asia Chemical Technology (Shandong) Co., Ltd. Urea (CO(NH_2_)_2_, AR) was obtained from Tianjin Ruijinte Chemical Co., Ltd. Titanium carbide (Ti_3_C_2_) monolayer nanosheet dispersion was obtained from Beijing Beike New Material Technology Co., Ltd. Potassium hydroxide (KOH, AR) was obtained from Sinopharm Chemical. Anhydrous ethanol (C_2_H_6_O, AR) was obtained from Chengdu Jinshan Chemical Reagent Co., Ltd. Nafion D-521 dispersion (5 %) was obtained from Alab (Shanghai) Chemical Technology Co., Ltd. Deionized water was used in all experiments. All chemicals and solvents were used without further purification.

### 2.2 Preparation of NiFe- LDH and NiFe-LDH/Ti_3_C_2_

NiFe-LDH sample was synthesized using hydrothermal method. First, 3.6 mmol Ni(NO_3_)_2_·6H_2_O, 1.8 mmol Fe(NO_3_)_3_·9H_2_O, 128 mmol CO(NH_2_)_2_ and 27 mmol NH_4_F were added into in 100 mL deionized water. The mixture was stirred at 25 °C for 1 h. Next, the solution was transferred into a 200 mL autoclave and heated at 105 °C for 12 h. Then, the products were naturally cooled to room temperature and washed three times with deionized water, collected by suction filtration, and placed in an electric air-drying oven for 12 h. Finally, the dried samples were ground into powder with a mortar for characterization.

The synthesis method of NiFe-LDH/Ti_3_C_2_ is similar to that of NiFe-LDH; an amount of Ti_3_C_2_ nanosheet dispersion solution was added during the solution preparation process of the NiFe-LDH. The addition amount of Ti_3_C_2_ was measured by weight, for example, 15 mg addition of Ti_3_C_2_ into the 100 mL solution was marked as NiFe-LDH/Ti_3_C_2_-15 mg, and so on.

### 2.3 Sample characterization

A D8 advance X-ray diffractometer (German Brooke Co.) with a Co Kα radiation source of λ = 1.79026 Å was used to examine the crystal structure of the as-prepared NiFe-LDH and NiFe-LDH/Ti_3_C_2_. A field emission scanning electron microscope (SEM, HITACHI SU8020) equipped with an energy dispersive X-ray detector (EDS) was employed to characterize the morphology and elemental composition of the as-prepared catalysts. The microstructure of the samples was probed by using transmission electron microscopy (TEM), which was recorded on an FEI Talos F200× electron microscope with an accelerating voltage of 200 kV. The surface chemical composition and the electronic structure of NiFe-LDH and NiFe-LDH/Ti_3_C_2_ were determined by using X-ray photoelectron spectroscopy (XPS), which was performed on an Escalab 250Xi electron spectrometer using a non-monochromatic Al Kα radiation (150 W) as the excitation source. The base pressure in the analysis chamber was about 2 × 10^−9^ mbar. All the XPS spectra were calibrated by adjusting the internal standard C 1s core level position to 284.8 eV. The elemental composition is quantitively measured by using inductively coupled plasma optical emission spectrometry (ICP-OES), which was performed on a ThermoICPOES 7200 with the radio frequency power of 1.15 kW and Argon carrier gas in axial mode detection.

### 2.4 Electrocatalytic performance testing

An electrochemical workstation (CHI650E) with a three-electrode system was used to perform all electrochemical performance tests in the OER process, and the electrolyte used a 1 M KOH solution. The counter electrode, reference electrode, and working electrode were the graphite rod, Hg/HgO electrode, and glassy carbon electrode, respectively. In all measurements, the potential values were changed to a reversible hydrogen electrode (RHE): E_RHE_ = E_Hg/HgO_ + 0.923 V. First, an activation scan of 20 circulations of cyclic voltammetry (CV) cycles was performed at 0.1 V s^−1^ to obtain stable curves. Then, the linear sweep voltammetry (LSV) was tested at 5 mV s^−1^ in the potential range of 0–0.8 V. The electrochemical impedance spectroscopy (EIS) tests were performed from 100 kHz to 1 Hz.

### 2.5 Computational details

All spin-polarized density-functional theory (DFT) computations were performed using the Vienna ab initio simulation package (VASP) based on the projector augmented wave (PAW) method. Electron-ion interactions were described using standard PAW potentials. A plane-wave basis set was employed to expand the smooth part of the wave functions with a cutoff kinetic energy of 400 eV. For the Electron-electron exchange and correlation interactions, the functional parametrized by Perdew-Burke-Ernzerhof (PBE), a form of the general gradient approximation (GGA), was used throughout. The van der Waals interaction was described via the DFT-D3BJ method.

To study the mechanistic chemistry of surface reactions, the surface was modelled with a slab model. A suffciently large vacuum region of 15 Å was used to ensure the periodic images were well separated. During the geometry optimizations, the bottom atoms were fixed at the bulk position when the surface properties were calculated. In this work, the Brillouin-zone integrations were conducted using Monkhorst-Pack grids of special points with a separation of 0.07 Å^−1^. The H_2_O and H_2_ molecules were calculated in a 10 × 10 × 10 Å^3^ box. The Brillouin-zone integrations were performed using the Gamma-point-only grid. The convergence criterion for the electronic self-consistent loop was set to 10^−5^ eV. The atomic structures were optimized until the residual forces were below 0.03 eV Å^−1^.

## 3 Results and discussion

### 3.1 Physical characterization of NiFe-LDH and its derivatives

**Fig. 1:**
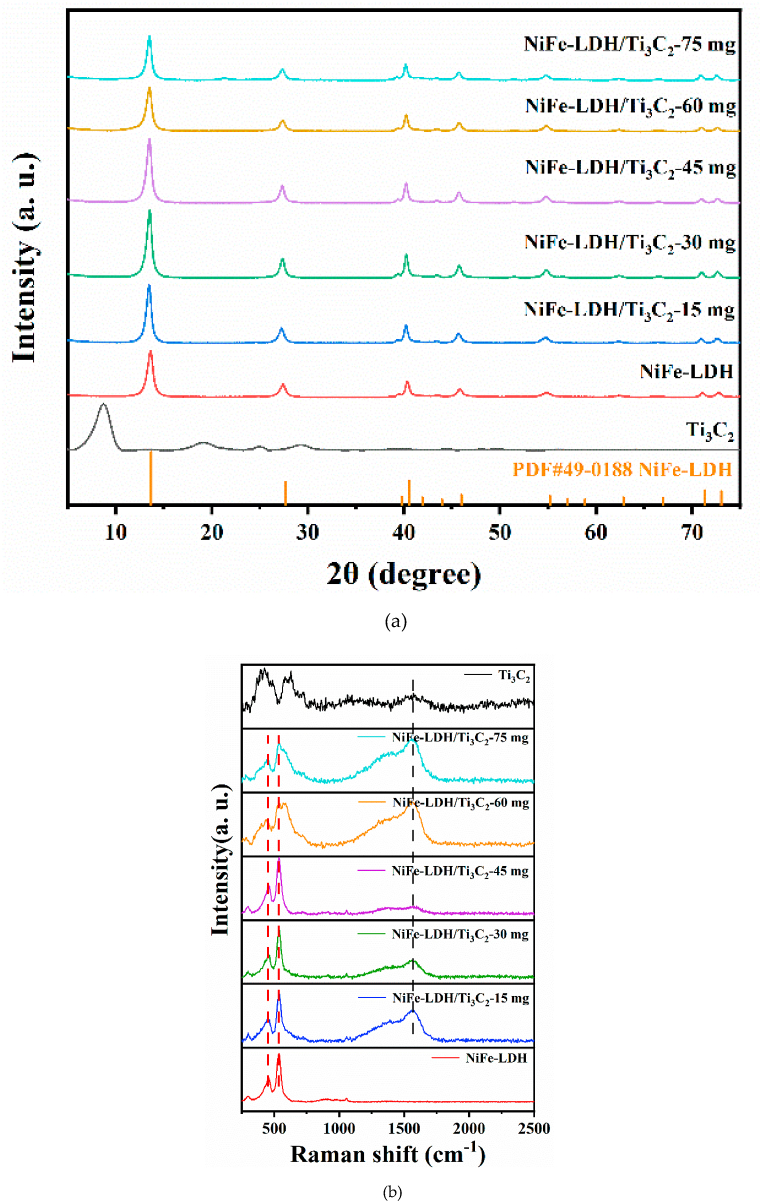
XRD (a) and Raman spectroscopy (b) of NiFe-LDH, Ti_3_C_2_, and NiFe-LDH/Ti_3_C_2_.

The crystal structure of NiFe-LDH, Ti_3_C_2_, and NiFe-LDH/Ti_3_C_2_ were analyzed using XRD. As shown in Fig. 1a, it is clear that the peak at 2θ of 9.05° is attributed to the (002) plane of Ti_3_C_2_. The diffraction peaks at 2θ = 13.7°, 27.7°, 40.6°, 46.0°, 55.2°, and 73.0° are well matched with the (003), (006), (012), (015), (018), and (113) diffraction planes of NiFe-LDH (JCPDS: #49-0188). However, for the synthesized NiFe-LDH/Ti_3_C_2_ composite, the characteristic peak of Ti_3_C_2_ (9.05°) did not show up because the proportion of Ti_3_C_2_ in NiFe-LDH/Ti_3_C_2_ was too low to be detected. Another reason is possible due to the preparation procedure that the NiFe-LDH was prepared as the reacting precursors were put together with the Ti_3_C_2_ material, that is NiFe-LDH was growing on the Ti_3_C_2_, therefore, Ti_3_C_2_ might be enveloped or encapsulated inside by the NiFe-LDH, and the signal of Ti_3_C_2_ is probably masked when the direct measurement of the surface of the samples. In order to confirm Ti_3_C_2_ was contained in the NiFe-LDH/Ti_3_C_2_ composite, Raman spectroscopy and ICP-OES has been conducted. In Fig. 1b, there was two obvious peaks at about 540 cm^−1^ and 450 cm^−1^ for the NiFe-LDH. Then, an obvious peak at about 1550 cm^−1^ was appeared in the NiFe-LDH/Ti_3_C_2_, corresponding to the characteristic peak of Ti_3_C_2_. This result suggested that the NiFe-LDH/Ti_3_C_2_ composite was successfully prepared. Moreover, ICP-OES measurements were carried out to evaluate the Ti element content in NiFe-LDH/Ti_3_C_2_ (Table S1 of the supplementary information). The content of Ti in NiFe-LDH/Ti_3_C_2_-60 mg was 3.44 wt%, which is lower to NiFe-LDH/Ti_3_C_2_-75 mg (4.28 wt%), but was higher than NiFe-LDH/Ti_3_C_2_-45 mg (2.65 wt%), NiFe-LDH/Ti_3_C_2_-30 mg (1.81 wt%), NiFe-LDH/Ti_3_C_2_-15 mg (0.75 wt%), and NiFe-LDH (0 wt%). The above results indicate that Ti_3_C_2_ was successfully doped in NiFe-LDH/Ti_3_C_2_ composite.

**Fig. 2:**
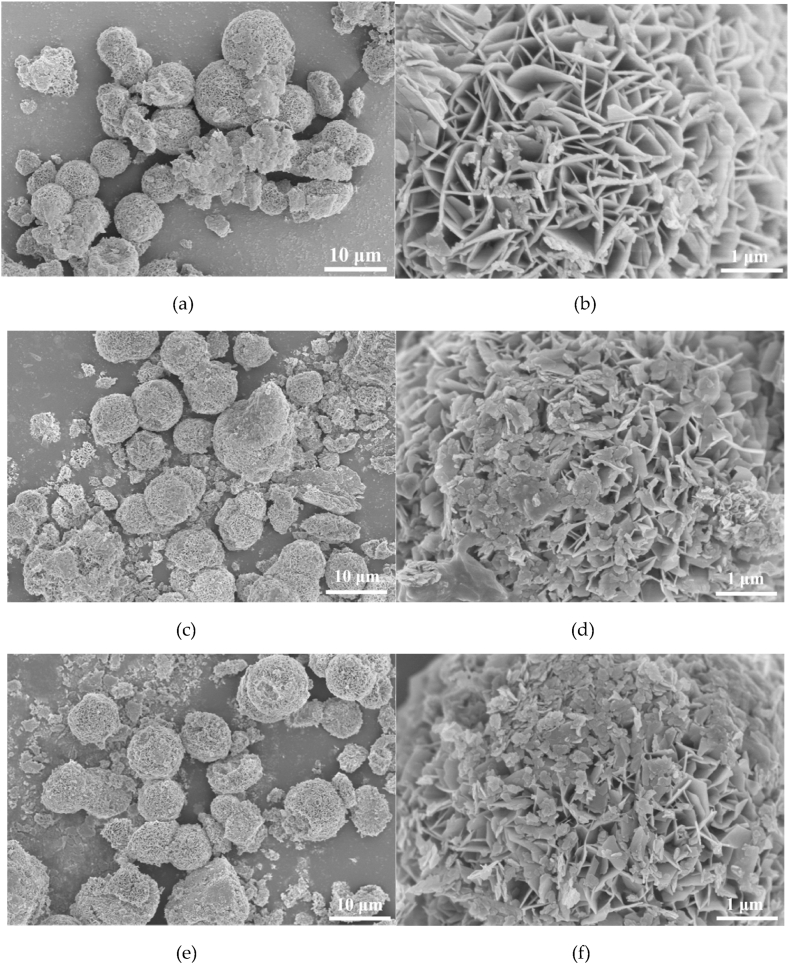

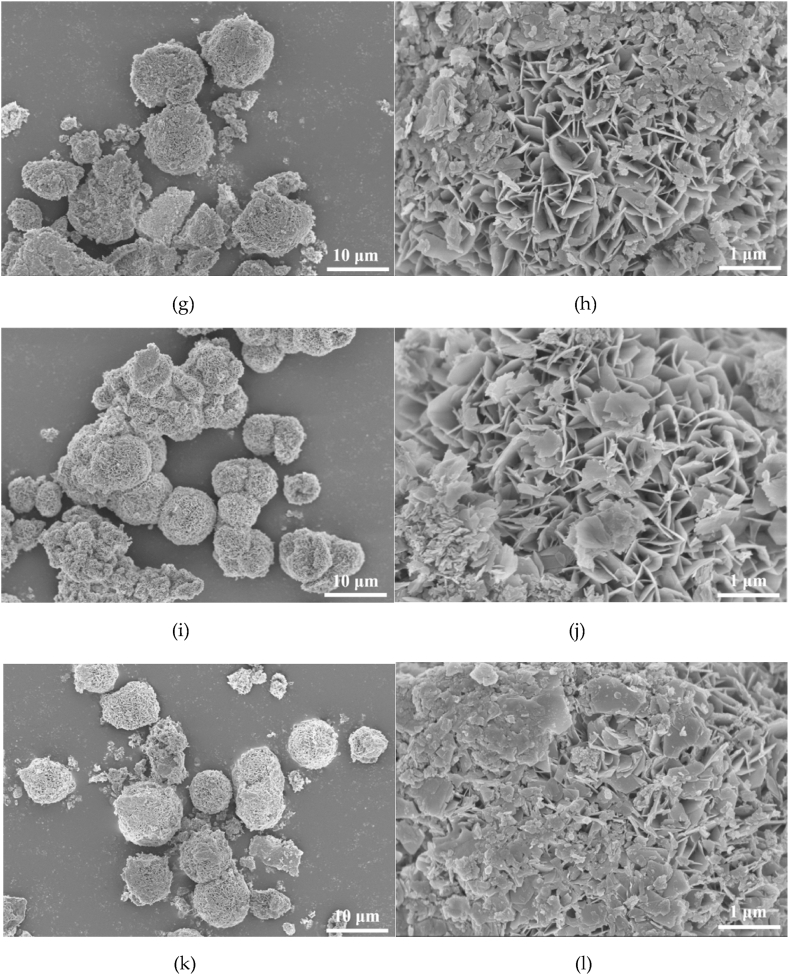
Scanning electron micrographs (SEM) of NiFe-LDH and NiFe-LDH/Ti_3_C_2_: (a, b) NiFe-LDH; (c, d) NiFe-LDH/Ti_3_C_2_-15 mg; (e, f) NiFe-LDH/Ti_3_C_2_-30 mg; (g, h) NiFe-LDH/Ti_3_C_2_-45 mg; (i, j) NiFe-LDH/Ti_3_C_2_-60 mg; and (k, l) NiFe-LDH/Ti_3_C_2_-75 mg.

The morphologies of NiFe-LDH and NiFe-LDH/Ti_3_C_2_ exhibited a flower-like structure assembled from the interconnected iron–nickel nanosheets with a thickness about 50 nm, and the average diameter of the nano-flower is approximately 6 μm (Fig. 2). The flower-like morphology with larger specific surface area and multilayer structure can largely accelerate the mass transfer, exposure of more active sites and provide larger reaction space.

**Fig. 3:**
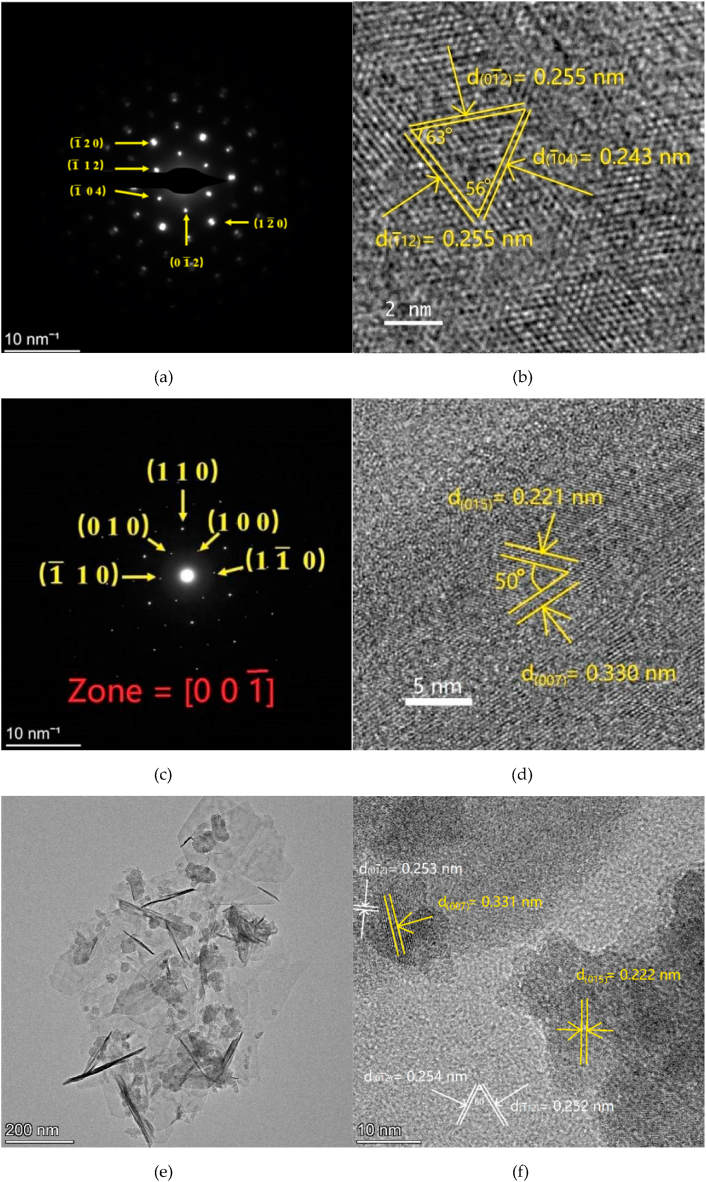
(a) SAED of NiFe-LDH; (b) HRTEM image of NiFe-LDH; (c) electron diffraction of Ti_3_C_2_; (d) high-resolution TEM of Ti_3_C_2_; (e) morphological magnification of NiFe-LDH/Ti_3_C_2_-60 mg; and (f) high-resolution TEM image of NiFe-LDH/Ti_3_C_2_-60 mg.

Fig. 3 reveals the TEM images of as-prepared NiFe-LDH sample. The selected area electron diffraction (SAED) of prepared NiFe-LDH shows a point diffraction pattern, which indicates that the prepared NiFe-LDH possesses excellent crystallinity (Fig. 3a). In Fig. 3b, the lattice distances of 0.255 nm, 0.243 nm, and 0.255 nm were attributed to the (0 $\bar{1}$ 2), ($\bar{1}$ 0 4), and ($\bar{1}$ 1 2) planes of NiFe-LDH, respectively. The angle between crystal lattices (0 $\bar{1}$ 2) and ($\bar{1}$ 1 2) is 63°, which matches well with the theoretically calculated value of 65°. The angle between crystal lattices ($\bar{1}$ 0 4) and ($\bar{1}$ 1 2) is 56°, which is in good agreement with the theoretical value of 57°. The measured crystal lattice distances and angles were highly consistent with the theoretically calculated crystal lattice distances and angles, which demonstrates that the prepared NiFe-LDH was a hexagonal crystal system. By calibrating the diffraction pattern, the Ti_3_C_2_ sample is a hexagonal crystalline system, which is consistent with these XRD results (Fig. 3c). From the high-resolution TEM, the lattice spacings of 0.221 nm and 0.330 nm were attributed to the (0 1 5) and (0 0 7) planes of Ti_3_C_2_. Fig. 3e and f shows the transmission electron micrographs of the prepared NiFe-LDH/Ti_3_C_2_ composite sample. The microscopic morphology of the prepared NiFe-LDH/Ti_3_C_2_ was approximately hexagonal in the field of view of 200 nm (Fig. 3e). In Fig. 3f, the lattice fringes with distances of 0.222 nm and 0.331 nm in the darker region are assigned to Ti_3_C_2_ (0 1 5) and (0 0 7) planes, respectively, while the lattice spacings of 0.254 nm and 0.252 nm in the lighter region matched well the crystallographic planes (0 $\bar{1}$ 2) and ($\bar{1}$ 1 2) of NiFe-LDH, respectively. Meanwhile, the angle between crystallographic planes (0 1 2) and ($\bar{1}$ 1 2) of NiFe-LDH is 60°, which further verifies the NiFe-LDH in the lighter region of the micrograph. Both Ti_3_C_2_ and NiFe-LDH lattice fringes are found in the micrograph confirms the formation of NiFe-LDH/Ti_3_C_2_ heterostructure composite and they are successfully combined together. The formation of NiFe-LDH/Ti_3_C_2_ heterojunction suggests that the Ti_3_C_2_ is successfully connected with the NiFe-LDH sheets, which would be beneficial to the composite in the electrical and electronic perspectives, as the Ti_3_C_2_ possesses a zero energy bandgap and exhibits a metallic conductivity arising mainly from the Ti 3d spin orbital according to the DFT theoretical calculations (see Fig. S1 in the supplementary information).

**Fig. 4:**
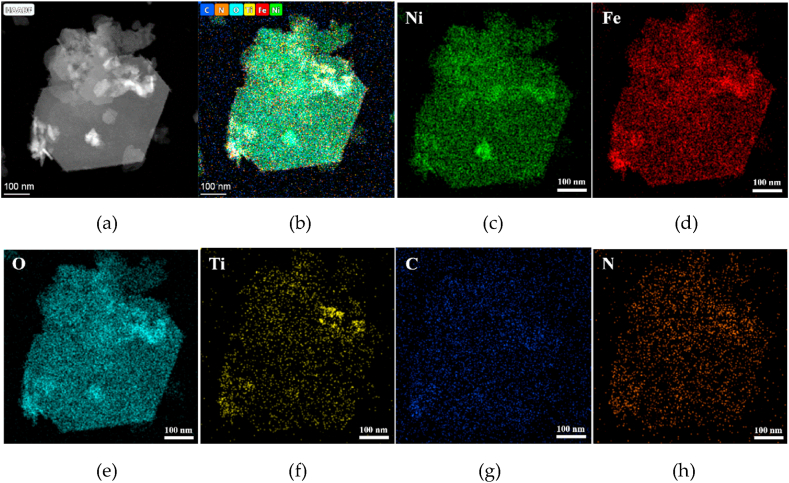
(a) HAADF image and (b)–(h) MAPPING image of NiFe-LDH/Ti_3_C_2_-60 mg.

Fig. 4a–h shows that the prepared NiFe-LDH/Ti_3_C_2_-60 mg contained six elements: Ni, Fe, O, Ti, C, and N. The content ratio of Ni, Fe, and Ti elements was approximately 6:4:1, which indicates that Ni and Fe had higher contents than Ti and the Ni-rich contents of the prepared layered double hydroxides. It is clearly seen that the Ni, Fe, and O are uniformly distributed in the main feature presented in Fig. 4a (HAADF), while the Ti distribution concentrates in the right corner of the feature, implying the combination of the Ti_3_C_2_ with NiFe-LDH in the composite.

**Fig. 5:**
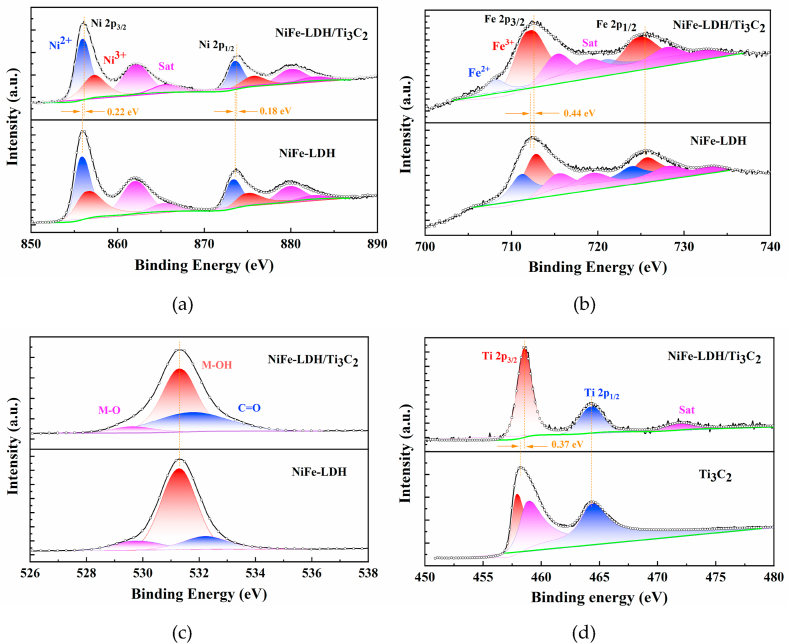
X-ray photoelectron spectra of (a) Ni 2p, (b) Fe 2p, (c) O 1s, and (d) Ti 2p for NiFe-LDH (lower) and NiFe LDH/Ti_3_C_2_-60 mg (upper).

Fig. 5 shows the XPS spectra of the composites NiFe-LDH/Ti_3_C_2_ and NiFe-LDH, and the results indicate that the composites contain elements of Ni, Fe, O, C, and Ti, which match the EDS results. It is worthy of noting that all the XPS spectra were calibrated by adjusting the internal standard C 1s core level position to 284.8 eV. The C 1s spectra for NiFe-LDH and NiFe-LDH/Ti_3_C_2_ are presented in the supplementary materials (Fig. S2). It is worthy of noting in Fig. S2 that when Ti_3_C_2_ is incorporated into NiFe-LDH, the intensity of C–*O*–C bonds is obviously increased compared to that of the pure NiFe-LDH, implying the bond interactions between the Ti_3_C_2_ and NiFe-LDH in the composite. The high-resolution Ni element (Fig. 5a) was divided into two separate peaks at 856.1 eV and 873.6 eV, ascribed to Ni 2p_3/2_ and Ni 2p_1/2_, respectively. Meanwhile, the fitting results show that Ni 2p_3/2_ and Ni 2p_1/2_ can be fitted to two peaks corresponding to Ni^2+^ and Ni^3+^. Both Ni 2p_3/2_ and Ni 2p_1/2_ are shifted in the direction of higher binding energy compared to the pure NiFe-LDH by 0.22 eV and 0.18 eV, indicating the valence state of Ni in the composite is increased than that of the pure NiFe-LDH. In the case of Fe 2p (Fig. 5b), Fe 2p_3/2_ orbitals shift positively by 0.44 eV, suggesting a higher valence state of Fe in the composite. Meanwhile, the peak area of Fe^3+^ in the fitting result is also larger, which can prove Fe element mainly exists in the form of Fe^3+^. It is reported that the high valence of Fe and Ni sites helps strengthen the binding with OH^−^ to promote the formation of the oxygen-containing intermediates (*OH, *O and *OOH), and thus accelerating the OER reaction kinetics [[30], [31], [32], [33]]. In Fig. 5c, the O 1s peaks can be divided into three separate peaks at 529.6 eV, 531.3 eV and 531.7 eV, which correspond to the “M–O”, “M–OH”, and “C]O” bonds, respectively [34]. From Fig. 5d, the two peaks at 458.6 eV and 464.2 eV in the Ti 2p spectrum are attributed to Ti 2p_3/2_ and Ti 2p_1/2_ and the peak at 471.8 eV is its corresponding satellite peak. The Ti 2p_3/2_ orbitals are shifted towards the direction of high binding energy compared to NiFe-LDH, so the valence state of Ti is elevated in the composite. The composite of two-dimensional materials increases the proportion of highly valent metals, which accelerates the rate of oxygen precipitation reaction. The variation of the NiFe-LDH/Ti_3_C_2_ valence states compared to the pure NiFe-LDH and pure Ti_3_C_2_ suggests a strong electronic coupling between the Ti_3_C_2_ and NiFe-LDH. The regulation of the electronic structure of NiFe-LDH by the Ti_3_C_2_ doping would provide a more optimal adsorption energy for the oxygen-containing intermediates during the OER process and accelerate the OER kinetics [[30], [31], [32], [33]].

### 3.2 Electrochemical performance of OER catalysts in an alkaline medium

**Fig. 6:**
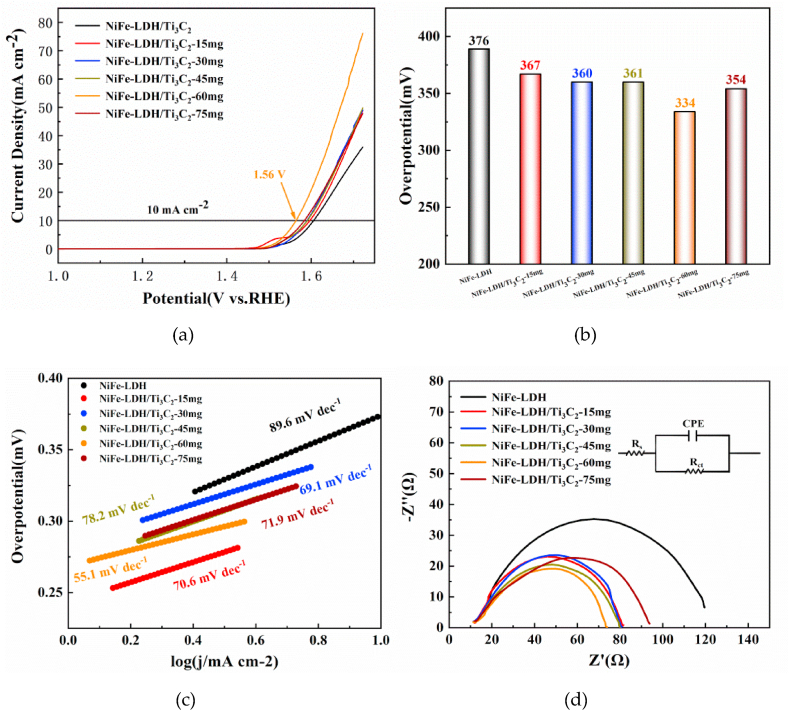
Linear voltametric curves of the prepared NiFe-LDH/Ti_3_C_2_ with different Ti_3_C_2_ addition amounts of 0 mg, 15 mg, 30 mg, 45 mg, 60 mg, 75 mg: (a) LSV curves; (b) overpotential; (c) Tafel slope; and (d) electrical impedance.

LSV polarization curves were used to compare their catalytic performance. In Fig. 6a, the voltage values of the six conditions were 1.606 V, 1.597 V, 1.590 V, 1.591 V, 1.564 V, and 1.584 V while delivering a current density of 10 mA/cm^2^, and the corresponding overpotentials were calculated to be 376 mV, 367 mV, 360 mV, 361 mV, 334 mV, and 354 mV (Fig. 6b), respectively. Thus, the electrochemical properties of the prepared NiFe-LDH/Ti_3_C_2_ with different Ti_3_C_2_ addition amounts gradually improved. The overpotential of NiFe-LDH/Ti_3_C_2_-60 mg was 334 mV, which demonstrates a 11 % reduction compared with the pristine. All NiFe-LDH/Ti_3_C_2_ samples exhibit lower overpotential than that of NiFe-LDH. Fig. 6c shows the Tafel slope plots of NiFe-LDH and NiFe-LDH/Ti_3_C_2_ for the samples with 89.6 mV/dec, 70.6 mV/dec, 69.1 mV/dec, 78.2 mV/dec, 55.1 mV/dec, and 71.9 mV/dec, respectively. Thus, the NiFe-LDH/Ti_3_C_2_-60 mg sample possesses the smallest Tafel slope of 55.1 mV/dec, which is 38.5 % lower than that of the pristine NiFe-LDH. All prepared NiFe-LDH/Ti_3_C_2_ samples exhibit smaller Tafel slopes than that of the pure NiFe-LDH samples. All samples with Ti_3_C_2_ doping present a lower overpotential and smaller Tafel slope compared to the pure NiFe-LDH and pure Ti_3_C_2_ (in Fig. S3 of the supplementary information), demonstrating the feasibility and effectiveness of regulating effect of 2D Ti_3_C_2_ on the NiFe-LDH electrocatalyst.

EIS was used to evaluate the catalyst conductivity. As shown in Fig. 6d, the electrical impedance of the pure NiFe-LDH is 110.2 Ω, while the NiFe-LDH/Ti_3_C_2_ with different addition amount are 71.8 Ω (15 mg addition), 69.2 Ω (30 mg), 68.3 Ω (45 mg), 59.6 Ω (60 mg), and 83.3 Ω (75 mg), respectively. It is obviously seen that the incorporation of Ti_3_C_2_ greatly reduces the charge transfer resistance and increases the electrical conductivity, thus promotes the OER activity. The synthesized NiFe-LDH/Ti_3_C_2_-60 mg had the smallest charge transfer resistance and the best electrical conductivity, which exhibits a 45.9 % improvement. NiFe-LDH and Ti_3_C_2_ forms a heterojunction, promoting the charge transfer and enhancing the conductivity of the composite, and consequently improving the electrochemical properties of the composite electrocatalyst.

**Fig. 7:**
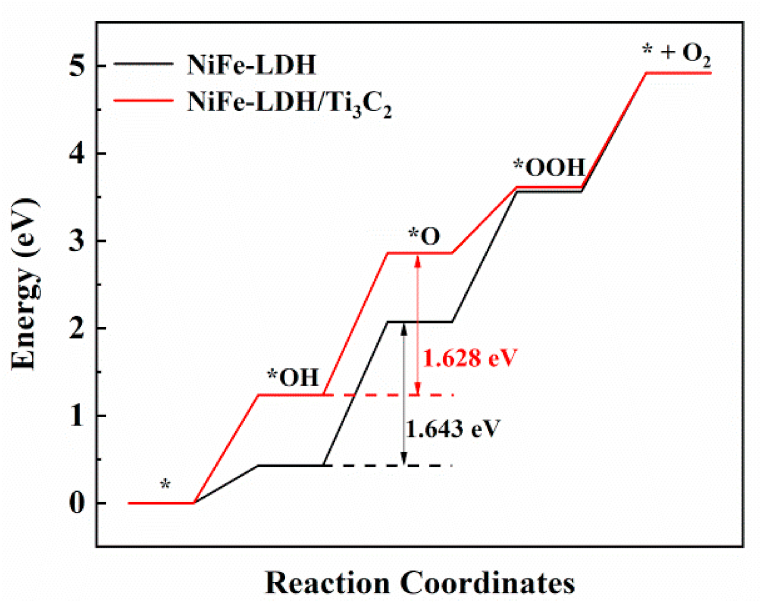
Free energy profile of the OER reaction path for two materials.

The free energy change during the OER four-electron transfer was calculated using a density functional theory calculation. The OER process involves four elementary reaction steps, in which *OH is formed from adsorbed OH^−^ and then is further oxidized to *O and *OOH. The corresponding adsorption free energy diagrams for the OER are shown in Fig. 7. The computations reveal that the Gibbs free energies of the four reactions for the pristine NiFe-LDH are calculated to be 0.429, 1.643, 1.492, and 1.357 eV, respectively. The maximized Gibbs free energy step at the equilibrium potential (U = 0 V) is defined as the rate-determining or rate-limiting step. Therefore, the highest barrier of the OER occurred at the transition of *OH to *O, which exhibited an energy difference of 1.643 eV and was considered the rate-determining step for pristine NiFe-LDH. In contrast, the free energy difference between the four steps for the NiFe-LDH/Ti_3_C_2_ is calculated as 1.234, 1.628, 0.752, and 1.306 eV, respectively. Therefore, the formation of *O is still the rate-limiting step for the NiFe-LDH/Ti_3_C_2_, which is slightly reduced compared to that of the pristine NiFe-LDH. By taking the OER equilibrium potential of 1.230 V into account, the theoretically calculated overpotential for the NiFe-LDH/Ti_3_C_2_ is determined to be 398 mV, close to the experimental results. The increased valence state of the Ni and Fe active sites induced by the incorporation of Ti_3_C_2_, could reduce the Gibbs free energy change of the reaction process and thus enhancing the catalytic activity [[30], [31], [32], [33]]. The theoretical calculation is consistent with the measured experimental results.

In this work, we focus on the decoration of NiFe-LDH by Ti_3_C_2_ doping in the electrical, electronic and energetic aspects. Obvious improvements in the electronic environment, charge transfer, and Gibbs free energy difference during the OER process are found, confirmed, and also can be well explained. Other factors may also have influences on the performance of the prepared electrocatalysts, such as the oxidation state of Ti_3_C_2_ throughout its lifetime, which will be investigated in-depth in the future work.

## 4 Conclusion

In this work, we aimed to design a low-cost and highly efficient OER electrocatalyst based on NiFe-LDHs. To overcome the disadvantages of poor electrical conductivity and low catalytic activity of NiFe-LDH, a kind of two-dimensional materials Ti_3_C_2_ was introduced. The Ti_3_C_2_ was successfully incorporated, bridging the nanosheets of NiFe-LDH to substantially improve the electrical conductivity by 45.9 %. Furthermore, Ti_3_C_2_ modulates the electronic environment of the NiFe-LDH, elevating the valence states of Ni and Fe active sites and thus bringing benefits to the OER activity. Theoretical calculations also reveal that the incorporation of Ti_3_C_2_ can effectively reduce the energy barriers during the reaction path, favoring the adsorption of the intermediates and promoting the OER process. Therefore, the prepared NiFe-LDH/Ti_3_C_2_-60mg composite electrocatalyst exhibits a low overpotential of 334 mV and a small Tafel slope of 55.1 mV dec^−1^, which is 11.2 % and 38.5 % lower than those of the pristine NiFe-LDH, respectively. This work provides an effective solution to design highly efficient electrocatalysts based on the non-noble metals.

## CRediT authorship contribution statement

**Yaxun Sun:** Writing – original draft, Methodology, Investigation, Data curation, Conceptualization. **Ze Wang:** Writing – original draft, Methodology, Data curation, Conceptualization. **Qianyu Zhou:** Validation, Formal analysis. **Xin Li:** Validation, Formal analysis. **Dongye Zhao:** Validation, Formal analysis. **Bo Ding:** Writing – review & editing, Supervision, Funding acquisition. **Shifeng Wang:** Writing – review & editing, Supervision, Project administration, Funding acquisition, Conceptualization.

## Declaration of competing interest

The authors declare that they have no known competing financial interests or personal relationships that could have appeared to influence the work reported in this paper.
