# Supplementary material for: Ti3C2 mediates the NiFe-LDH layered electrocatalyst to enhance the OER performance for water splitting
Source: Heliyon. 2024 May 14;10(10):e30966. doi: 10.1016/j.heliyon.2024.e30966 (PMC11112313; doi:10.1016/j.heliyon.2024.e30966)
Supplement: Multimedia component 1 [file mmc1.doc]

Ti_3_C_2_ Mediates the NiFe-LDH Layered Electrocatalyst to Enhance the OER Performance for Water Splitting

Yaxun Sun, ^1,3^ Ze Wang, ^1,3^ Qianyu Zhou, ^1^ Xin Li, ^1^ Dongye Zhao, ^1^ Bo Ding, ^2,*^ and Shifeng Wang, ^1,*^

**Supplementary Information**

**S1:** Theoretical calculations of Ti_3_C_2_ electronic structure. It is observed that the band gap is zero (Figure S1a), suggesting a metallic conductivity theoretically speaking. The superior conductivity arises from the high density of state, which is contributed mostly by the Ti 3d spin orbital (Figure S1b).


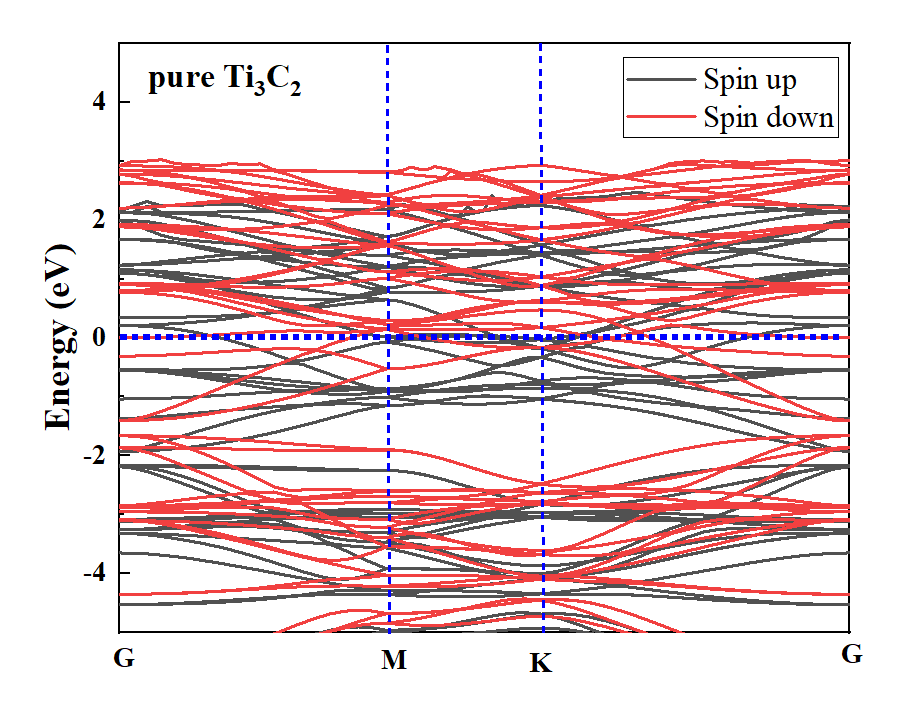

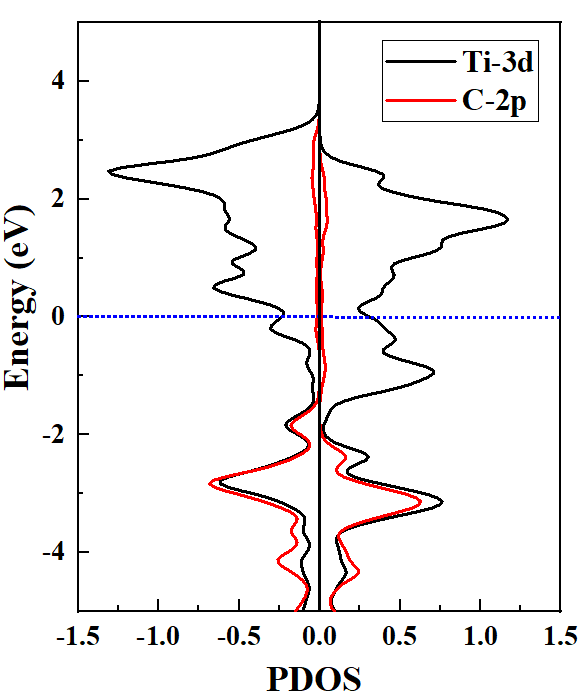


1. (b)

**Figure S1.** The electronic structure of Ti_3_C_2_ using DFT calculations. (a) Energy band gap; (b) Projected density of state (PDOS).

**Table S1: The ICP test of Sample.**

| **Sample** | **Element/Wt %** | | |
| --- | --- | --- | --- |
|  | **Ni** | **Fe** | **Ti** |
| NiFe-LDH | 40.73 | 14.37 | 0.00 |
| NiFe-LDH/Ti_3_C_2_-15 mg | 40.95 | 12.89 | 0.75 |
| NiFe-LDH/Ti_3_C_2_-30 mg | 39.57 | 11.90 | 1.81 |
| NiFe-LDH/Ti_3_C_2_-45 mg | 37.57 | 11.30 | 2.65 |
| NiFe-LDH/Ti_3_C_2_-60 mg | 38.86 | 11.72 | 3.44 |
| NiFe-LDH/Ti_3_C_2_-75 mg | 37.05 | 11.07 | 4.28 |

**S2:** XPS spectrum of C 1s of the samples. It is worthy of noting that when Ti_3_C_2_ is incorporated into NiFe-LDH, the intensity of C-O-C bonds is obviously increased compared to that of the pure NiFe-LDH, implying the bond interactions between the Ti_3_C_2_ and NiFe-LDH in the composite.


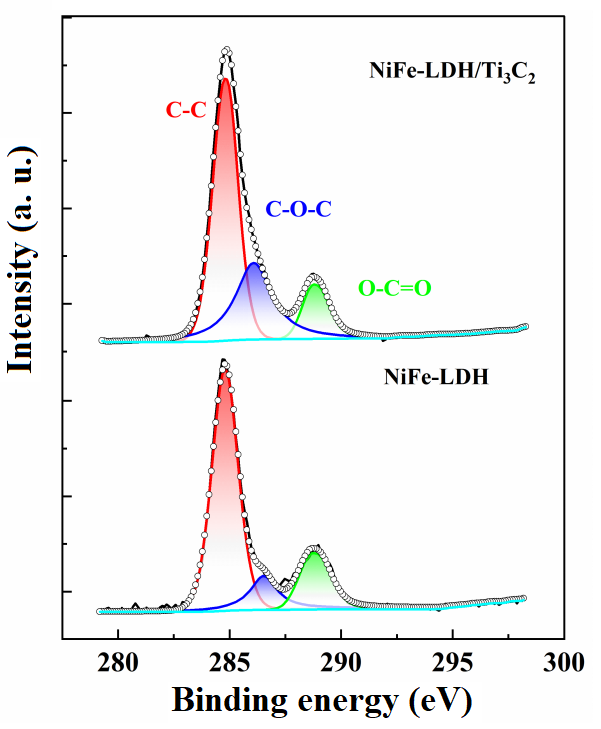


**Figure S2.** High-resolution XPS spectrum of C 1s for NiFe-LDH (lower) and NiFe LDH/Ti_3_C_2_ (upper).

**S3:** OER performance of Ti_3_C_2_. It is observed that the OER performance of pure Ti_3_C_2_ is rather poor. Therefore, the contribution of OER activity in the composite mostly relies on the NiFe-LDH.


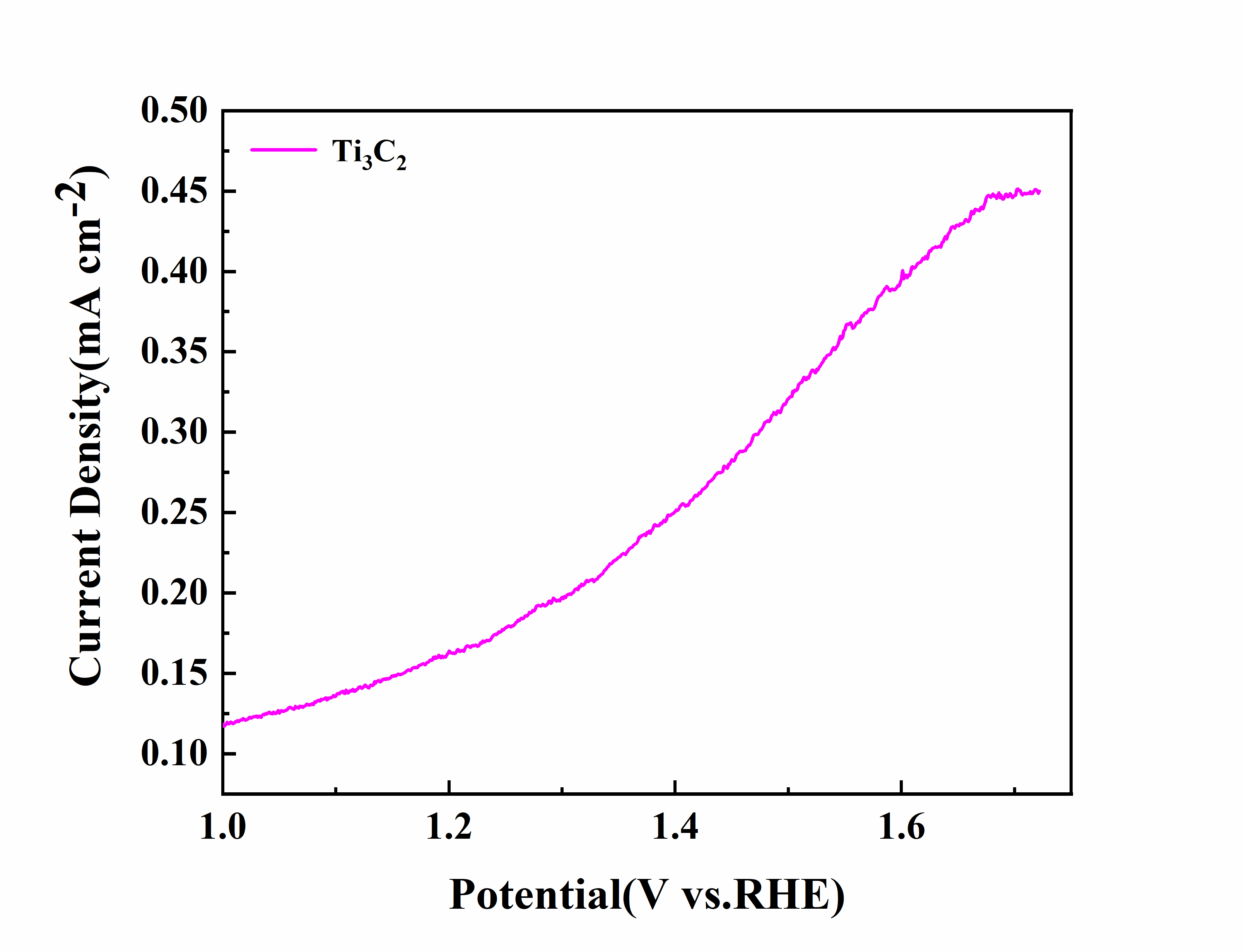

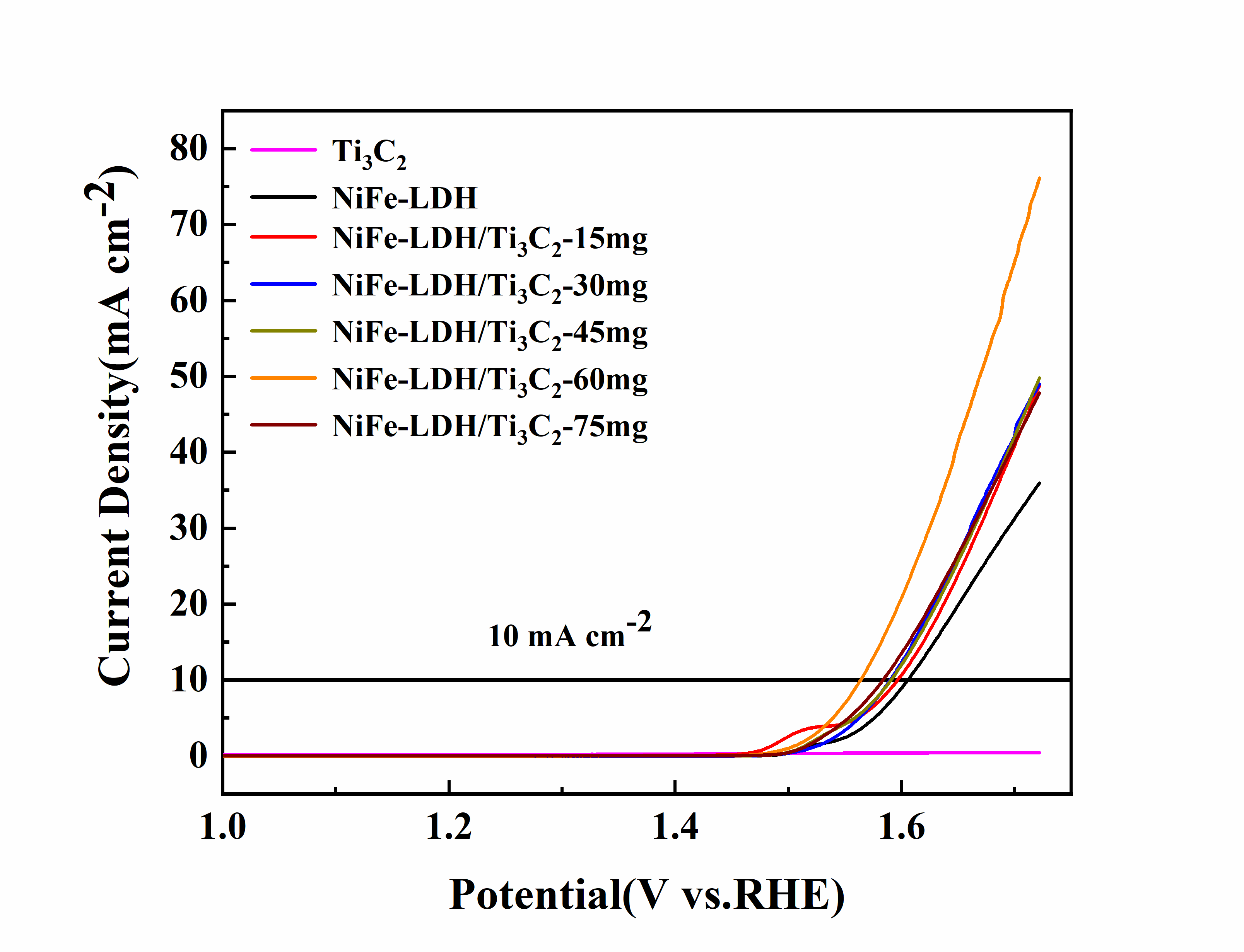


**Figure S3.** Comparative LSV plots of Ti_3_C_2_ alone and composite NiFe-LDH/Ti_3_C_2_.
